# *Fructus mume* Extracts Alleviate Diarrhea in Breast Cancer Patients Receiving the Combination Therapy of Lapatinib and Capecitabine

**DOI:** 10.3389/fphar.2018.00516

**Published:** 2018-05-23

**Authors:** Hua Xing, Lirong Zhang, Jinshu Ma, Zhen Liu, Changlong Song, Yuxia Liu

**Affiliations:** ^1^Department of Breast Surgery, China-Japan Union Hospital of Jilin University, Changchun, China; ^2^Department of Pathology, China-Japan Union Hospital of Jilin University, Changchun, China; ^3^Pediatrics, Liuhe District Hospital of Nanjing, Nanjing, China; ^4^Archives Center, China-Japan Union Hospital of Jilin University, Changchun, China

**Keywords:** lapatinib, capecitabine, diarrhea, risk, breast cancer, *Fructus mume*

## Abstract

Lapatinib and capecitabine have been widely used in the therapy of breast cancer. However, long-term use of lapatinib and capecitabine often causes the most common side effect diarrhea, which limit the medicine use. *Fructus mume (F. mume)* has been proved to be effective to treat chronic diarrhea with few side effects. The compounds from *F. mume* were extracted by using an ethanol method. Extracts of *F. mume* (EFM) were analyzed by HPLC. We investigated the protective effects of EFM on the diarrhea caused by lapatinib and capecitabine. From March 1st, 2016 to June 1st, 2017, 208 breast cancer patients with diarrhea caused by lapatinib and capecitabine were recruited. The patients were evenly assigned into two groups: EG group (the patients took 100 mg EFM daily) and CG group (the patients took placebo daily). The effects of EFM on diarrhea and gastrointestinal symptoms were measured by a semiquantitative method seven-point Likert scale. Overall quality of life was measured by SF-36 questionnaire and Hospital Anxiety and Depression Scale (HADS). The HPLC analysis showed that there were three components in EFM, including citric acid, 5-hydroxymethylfurfural (5-HMF), and chlorogenic acid. Breast cancer types were observed by using Hematoxylin and eosin (H&E) stain. The breast cancer can be divided into leaflet, gland and fibroblast types. Patient age, skin metastases, treatment, and grade 1 diarrhea were significant risk factors associated with for grade 2 diarrhea. EFM reduced diarrhea and gastrointestinal symptoms by reducing the average scores of the diarrhea symptom and seven-point Likert scale, and improved life quality of patients significantly by improving SF-36 scores and reducing HADS scores when compared to that in the CG group after 6-week therapy and further 4-week follow-up (*P* < 0.05). EFM may be a potential choice for the diarrhea therapy in breast cancer patients.

## Introduction

Lapatinib is an oral administration drug for the therapy of metastatic breast cancer (MBC) as a tyrosine kinase inhibitor (TKI) of the epidermal growth factor receptor (Lin et al., 2015). The combination of lapatinib and refametinib improves the symptoms of the patients with HER2-positive breast cancer. Lapatinib is effective in treating HER2-positive MBC, but it can cause serious side effects, particularly diarrhea (Dang et al., 2010; Dranitsaris and Lacouture, 2014) and skin rash (Parham et al., 2015; Sonnenblick et al., 2016). Capecitabine is another oral drug used in the therapy of breast cancer. The antitumor activity of capecitabine has been widely proved in the salvage therapy of breast cancer (Li Y.S. et al., 2017) and early breast cancer (EBC). However, capecitabine may cause hand-foot syndrome (Scontre et al., 2017; Singh et al., 2018), stomatitis (Mignogna et al., 2009), and diarrhea (Dranitsaris and Lacouture, 2014).

The combination of capecitabine and an anthracycline-taxane based regimen may be effective and well-tolerated EBC patients, especially for triple negative breast cancer (TNBC) (Zhang et al., 2017). HER2-positive breast cancer occupies about 20–25% of cases of breast cancer. Trastuzumab is usually considered in the first-line treatment. However, due to cardiotoxicity and increasing resistance associated with trastuzumab, it is highly demanded to explore an effective method for the therapy of breast cancer. There has been increasing interest in treating HER2-positive breast cancer by combining lapatinib and capecitabine. The results suggest that the combination of lapatinib plus capecitabine can improve progression-free survival and overall survival in patients with HER2-positive breast cancer that cannot be controlled by trastuzumab (Madden et al., 2017).

Diarrhea will be a main side effect since both lapatinib and capecitabine can cause diarrhea. Moderate to severe diarrhea (an increase of four to six stools over baseline daily) was reported in 20% of patients’ in the pivotal trial (Geyer et al., 2006). Diarrhea can result in dose reduction, delays, poor life quality, high cost for health care, and even life threatening complications sometimes (Mehmood et al., 2017).

Lapatinib and capecitabine therapy can induce mucosal barrier injury (Al-Dasooqi et al., 2013), which will result in bacterial infection (Blijlevens and Donnelly, 2011) and inflammatory responses (Blijlevens et al., 2000). All these results will contribute to the diarrhea occurrence. To reduce the side effects, natural products may be a potential option with limited side effects. *Fructus mume (F. mume)*, has long been proved to have anti-bacterial (Lee and Stein, 2011) and anti-inflammatory properties by reducing the levels of proinflammatory cytokines, angiotensin II (Ang II), the receptor for advanced glycation end-products (RAGE), and the mitogen-activated protein kinases (MAPKs) (Kim et al., 2016). *F. mume* has long been used in Asian countries to treat chronic diarrhea via its anti-inflammatory activities (Kim et al., 2016). An earlier study showed that *F. mume* could alleviate gastrointestinal diseases by down-regulating Th1-polarized immune response and opsonic effect of intestinal commensal bacteria (Liu et al., 2009). However, the effects of *F. mume* on human diarrhea are seldom reported. *F. mume* may be effective to treat diarrhea in breast cancer patients receiving the combination therapy of lapatinib and capecitabine, and related work was explored here.

## Materials and Methods

### Preparation of *F. mume* Extracts

*Fructus mume* was purchased from Chengdu Dujiangyan Chunsheng Chinese Medicine Drinks Co., Ltd. (Chengdu, China). The species was identified by Professor Bo Liu from Changchun University of Chinese Medicine (Changchun, China). *F. mume* was dried and extracted in 75% ethanol in a shake incubator (Beijing JinMei Entrepreneur Import & Export Co., Ltd., Beijing, China) overnight at room temperature and filtrated with Whatman Quantitative Filter Paper. The extracts of *F. mume* (EFM) were concentrated by using a rotary vacuum evaporator from Kelong Company (Beijing, China).

### HPLC Analysis

The following chromatographic condition was used: XBridge TMC18 column (4.6 mm × 250 mm, 5 μm); injection volume, 10 μl; detection wavelength, 210, 284, and 327 nm; mobile phase, (A) 0.5% NH_4_H_2_PO_3_ solution (phosphoric acid pH 3.0) – (B) acetonitrile gradient solution, 0–10 min, 95–80% A; 10–15 min, 80–70% A; 15–16 min 70–95% A; and 16–21 min, 95% A, 1 ml/min. Mixed solution of citric acid, 5-HMF, and chlorogenic acid standards (purchased from Sigma) was placed in a 5 mL flask, dissolved and fixed with 20% methanol. A series of control solution was made as the concentrations of 16,200.00, 6,400, and 2,400 mg/l. EFM was dried at low temperature and weighed 0.20 g, and placed in a 250-ml round-bottom flask. The solution was filtered by a 0.22 μm membrane, and diluted into 1 ml by adding 20% methanol.

Ten-microliters standard and sample solution was taken and analyzed according to above chromatographic condition. The detection wavelength was given as follows: citric acid (210 nm), 5-HMF (284 nm), and chlorogenic acid (327 nm). The mixed standards were sequentially diluted to obtain a series of standard solution: citric acid, 8,100.00, 4,050.00, 2,205.00, 1,012.50, 506.30, 253.10, 126.60, and 63.30 mg/l; 5-HMF, 32.00, 16.00, 8.00, 4.00, 2.00, 1.00, and 0. 50 mg/l; and chlorogenic acid, 12.00, 6.00, 3.00, 1.50, 0.75, 0.38, and 0.19 mg/l. The peak area integral value (*Y*) was designed as the ordinate, and the corresponding standard concentration was designed as abscissa (*X*). Linear regression equation was made finally.

### Analysis for the Ingredients of EFM From Different Batches

Nine batches of EFM powder (batch number 160903452) were prepared in accordance with the above method and analyzed by using above chromatographic condition. Ten-microliters samples were injected and peak areas of three components were measured. The contents of citric acid, 5-HMF, and chlorogenic acid were calculated.

### Patients

Before the experiments, all procedures were approved by the research ethical committee from Jilin University (Changchun, China). Meanwhile, written informed consent was obtained from the participants of this study. Clinical characteristics were analyzed, including demographic information, age, BMI (body mass index), and life style so on. In the present study, all patients previously received capecitabine (1.5 g/m^2^ daily every 14 days for one through 10 of a 3-week cycle) and oral lapatinib 1 g daily.

### Inclusion Criteria

The following inclusion criteria were used: occurrence of diarrhea was evaluated by asking during one cycle of combination therapy of lapatinib and capecitabine (at least 21 days), and all patients had more than one risk for ≥ grade 2 diarrhea (Increase of 4–6 stools daily over baseline.) because of the combination therapy of lapatinib and capecitabine; All patients were with advanced MBC.

### Exclusion Criteria

The following criteria was used: the patients suffered from heart [determined by cardiovascular questionnaire and electrocardiogram (ECG)], stomach (determined by stomach questionnaire and endoscopy), liver (determined by liver disease questionnaire and salt activity >50 U/L) and gallbladder (diagnosed by ultrasonic or X-ray techniques), pancreas (diagnosed by duodenoscopy) and kidneys (determined by ultrasound) diseases, and all diseases were determined by responding experts; The following methods were used to evaluate the organs dysfunction. A “threshold” serum creatinine value of 130 μmol/L serum creatinine was used to evaluate kidney dysfunction in women (Ali et al., 2007). Threshold of ALT and AST for the diagnosis of liver disease was 19.0 IU/L (Sahdev et al., 1991; Tomizawa et al., 2014). The threshold for GGT was 165 U/L for the evaluation of liver function (Guiu et al., 2012). The threshold for bilirubin toxicity was defined as 20 mg/dL for evaluation kidney function (Valaes, 1992). The threshold for lipase was 1000 U/L for the evaluation of pancreas function (Facy et al., 2012).

The patients had colonoscopy intestinal or esophageal stricture, gastrointestinal cancer and other gastrointestinal organic diseases; the patients were gestational or lactating women; the patients suffered from nervous system or mental illness, understanding obstacles, who cannot be communicated; the patients had past history of traditional Chinese medicine allergy. The patients took the treatment of this disease or other drugs affecting gastrointestinal function. The patients with stomas were excluded from the present study.

### Patients Grouping

The random numbers were generated by a computer combining with clinical variables According to the randomized controlled design method, all the patients were divided into treatment group (EG, the patients received 100 mg EFM daily) and control group (the patients received placebo daily) according to the patient’s selection time and random numbers. The chemotherapy was still continued during the whole present experiment. All patients from two groups have light or no work to avoid emotional stress or over exertion. Work has been reported to be associated with breast cancer progression (Mehmood et al., 2017) and diarrhea (Thorn and Kerekes, 2001). ‘Light’ work was defined as flexed postures, walking and repeated movements, which are involved with the upper limb and light weights (Al-Dasooqi et al., 2013). All patients received high protein, low fat and high fiber diet, and limited spicy irritating or greasy food. All the patients received the therapy for 6 weeks. After the therapy, further 4-week follow-up was performed to explore the long-term effects of EFM on diarrhea. Meanwhile, clinical symptoms were measured after 6-week therapy and further 4-week follow-up.

### Hematoxylin and Eosin (H&E) Stain

Five-milligram breast cancer specimens were obtained by needle biopsy. All manipulations of tissue isolation were performed in a biohazard safety laboratory (P3). The isolated specimens were immediately fixed in 10% formalin solution and embedded in paraffin. The embedded paraffin blocks were cut to a thickness of 4-μm section. H&E stain was performed according to an earlier report (Soares et al., 2007).

### Assessment of Diarrhea and Gastrointestinal Symptoms

Diarrhea symptom was scored according to an earlier report (Hart and Dobb, 1988). Briefly, a semiquantitative method to measure diarrhea scores. Three fecal volumes (+ < 200 mL, ++ 200–250 mL, +++ > 250 mL) and three kinds of fecal consistency [formed (scores, 1, 2, and 3), semisolid (scores, 3, 6, and 9), liquid (scores, 5, 15, and 15)]. Daily scores were calculated by summing the scores of all stools during a day. Gastrointestinal symptoms were measured according to abdominal Seven-point Likert scale (abdominal pain, bloating, straining, mucus, incomplete evacuation, urgency, wind, hard stool, loose stool, frequency of motions, and nausea) (Guthrie et al., 2003). Scores were recorded by the same corresponding experts during the while experiment. The study was continued for a maximum of 21 days for each time.

### Assessment of Quality of Life

Overall quality of life was measured by SF-36 questionnaire (Ware and Gandek, 1998) and Hospital Anxiety and Depression Scale (HADS) score (Ayis et al., 2018).

### Statistical Analysis

All statistical analyses were performed by using SPSS 20.0 (SPSS Inc., Chicago, IL, United States). All variables were expressed as mean ± standard deviation (SD). The relationship between clinical characters and diarrhea incidence was analyzed by using univariate and logistic regression. The significant variables (more than 10%) were included as possible indicators. A multivariate analysis was performed by using all the predictors who came out to be significant in the univariate analysis. Patient/treatment characteristics were compared to verify how well the patients were randomized. Chi squared test or student’s test was used to compare the treatment efficacy groups among CG and EG. Univariate analysis was performed to explore potential predictor factors associated with the diarrhea. The statistical differences were significant if *P* < 0.05.

## Results

### Main Ingredients of EFM

**FIGURE 1:**
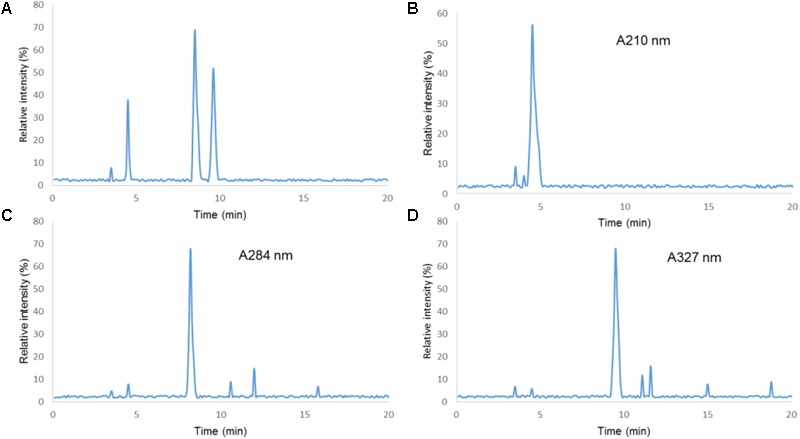
HPLC chromatograms of Extracts of *Fructus mume* (EFM) sample and standards. (1) Citric acid; (2) 5-HMF; (3) chlorogenic acid; **(A)** standard. **(B)** Sample detection at 210 nm. **(C)** Sample detection at 280 nm. **(D)** Sample detection at 327 nm.

The HPLC analysis showed that there were three components in EFM, including citric acid, 5-HMF, and chlorogenic acid (**Figure 1**). **Table 1** showed linear relationships of major components of EFM during HPLC analysis. Under the experimental condition, the theoretical area for the absorption peak of the chromatogram of the test sample and each standard was not less than 3,000, and the corresponding chromatographic peak resolution was >1.5.

**Table 1:** Linear relationships of major components of EFM.

Main components	Regression equation	*R*	Linear range	Measure limitation, mg/l	Quantitative limitation, mg/l
Citric acid	*Y* = 681.4X+30,313	0.9999	63.30 ~ 8,100.00	0.120	0.401
5-HMF	*Y* = 117,290X-72,086	0.9999	0.51 ~ 32.00	0.021	0.070
Chlorogenic acid	*Y* = 48,662-1300.4	0.9998	0.19 ~ 12.00	0.046	0.092

### The Contents of the Ingredients of EFM Were Stable at Different Batches

According to the above HPLC condition, the ingredients of EFM were measured nine times. The RSDs of the peak areas of the components, the peak areas of the obtained citric acid, 5-HMF, and the chlorogenic acid were 1.6, 2.1, and 1.5%, respectively. It indicated that the contents of the ingredients of EFM were stable as **Table 2** showed. Thus, EFM from different batches would not affect subsequent therapeutic results.

**Table 2:** HPLC analysis of EFM at different batches.

Ingredients	Total weight/g	Ingredient weight/mg	Addition/mg	Measurement/mg	Recovery/%	Mean value/%	RSD/%
Citric acid	0.200	63.389	50.708	114.092	99.308	99.952	1.632
	0.200	63.448	50.647	113.202	97.231		
	0.200	63.310	50.734	113.623	98.902		
	0.200	63.426	63.407	125.813	98.415		
	0.200	63.402	63.376	125.770	98.170		
	0.200	63.316	63.369	126.897	100.106		
	0.200	63.362	76.083	139.527	99.947		
	0.200	63.369	76.098	139.352	99.879		
	0.200	63.383	76.115	140.162	100.775		
5-HMF	0.200	0.367	0.264	0.607	98.702	99.136	1.5372
	0.200	0.324	0.231	0.696	100.921		
	0.200	0.321	0.220	0.674	100.270		
	0.200	0.389	0.336	0.767	98.203		
	0.200	0.343	0.345	0.744	97.159		
	0.200	0.383	0.337	0.786	100.491		
	0.200	0.358	0.401	0.808	98.664		
	0.200	0.348	0.409	0.840	99.504		
	0.200	0.329	0.440	0.851	97.825		
Chlorogenic acid	0.200	0.364	0.255	0.580	97.298	99.375	1.124
	0.200	0.364	0.238	0.579	100.926		
	0.200	0.299	0.276	0.628	98.587		
	0.200	0.281	0.316	0.706	98.625		
	0.200	0.350	0.322	0.697	98.856		
	0.200	0.332	0.318	0.722	100.187		
	0.200	0.343	0.365	0.758	99.690		
	0.200	0.271	0.383	0.780	99.294		
	0.200	0.353	0.417	0.804	100.396		

### Clinical Characteristic

**Table 3** showed the clinical characteristics of patients in both groups. Patients who received the combination therapy of lapatinib and capecitabine had a median age of 51 years, and over 90% had a good result. The combination therapy of lapatinib and capecitabine were delivered over a 4-month ere delivered over a 4-month period, and 208 patients had diarrhea episodes. Approximately, 23 and 18% of patients began to receive the therapy in spring and summer in EG and CG, respectively. With respect to the primary endpoint, 208 patients had ≥ grade 2 diarrhea episodes. The statistical difference for the numbers for light work was insignificant between two groups. Diet was controlled throughout the experiment period to match habitual intake according to **Table 3**.

**Table 3:** Characteristics of patients and treatment received.

Characteristic	EG (*n* = 104)	CG (*n* = 104)	*T* value and χ^2^	*P*-value
Age, year (range)	51 (25–79)	50 (28–78)	1.578	0.139^a^
BMI, kg/m^2^	23.4 ± 3.7	23.8 ± 4.2	0.287	0.453^a^
Smoking, *n* (%)	31	35	0.355	0.551^b^
Drinking, *n* (%)	28	24	0.410	0.522^b^
Light work, *n* (%)	78	83	0.687	0.407
**ECOG performance status**	
0	62	60	0.137	0.933^b^
1	37	38		
Missing	5	6		
Mean baseline Hb [g/dL] (SD)	12.4 ± 1.7	13.7 ± 1.9	-2.449	0.070^a^
Mean baseline WBC [×10^9^ cells/l]	5.9 ± 2.3	5.6 ± 2.0	1.264	0.274^a^
Mean baseline platelets [×10^9^cells/l]	243 ± 80	235 ± 86	1.568	0.164^a^
Median number of metastatic sites	4 ± 3	4 ± 3	0.316	0.767^b^
Surgical treatment				0.497	0.780^b^
Lumpectomy, *n* (%)	41	46		
MRM, *n* (%)	62	57		
Both lumpectomy and MRM, *n* (%)	1	1		
Radiation				0.079	0.961^b^
Left sided, *n* (%)	57	59		
Right sided, *n* (%)	46	44		
Bilateral, *n* (%)	1	1		
**Metastatic sites**	
Liver	54	52	0.108	0.998^b^
Bone	46	45		
Lung	44	46		
Skin	19	18		
Brain	4	4		
Total number of cycles delivered	1,321	1,302		
Median number of cycles	18 ± 15	18 ± 15	0.244	0.451^a^
Grade 1 diarrhea in the prior cycle	104 events	104 events		
Concomitant 5HT3 antiemetics	In 15 cycles	In 15 cycles		
**Season treatment was started**	
Spring	24	26	0.048	0.997^b^
Summer	18	17		
Fall	30	31		
Winter	32	32		
Development of grade ≥2 diarrhea	59 events at median of six, 21-day cycle	56 events at median of six, 21-day cycle	0.175	0.675^b^
**Food categories (g/day)**	
Starchy foods (breads, cookies, potato, rice)	102.4 ± 15.7	96.8 ± 13.4	0.764	0.249
Legumes (chick peas, beans, peas, lentils)	28.4 ± 5.9	29.1 ± 6.3	0.453	0.518
Vegetables (pumpkin, chayote, tomato, carrot)	358.9 ± 124.7	309.5 ± 118.4	0.795	0.127
Meat, poultry, fish and eggs	89.7 ± 40.3	98.5 ± 36.2	0.681	0.426
Fruits	146.3 ± 52.4	156.1 ± 61.3	0.328	0.593
Dairy products (ml)	150.0 ± 50.0	150.0 ± 50.0	0.148	0.867
Sweet foods (sweet guava, ice cream, dulce de leche)	78.5 ± 45.2	80.1 ± 41.6	0.115	0.859
Spicy foods (ketchup, pepper)	15.4 ± 6.8	14.9 ± 7.6	0.147	0.682

*ECOG, Eastern Cooperative Oncology Group; ^*a*^T-test and ^*b*^Chi Square*.

### Breast Cancer Types

**FIGURE 2:**
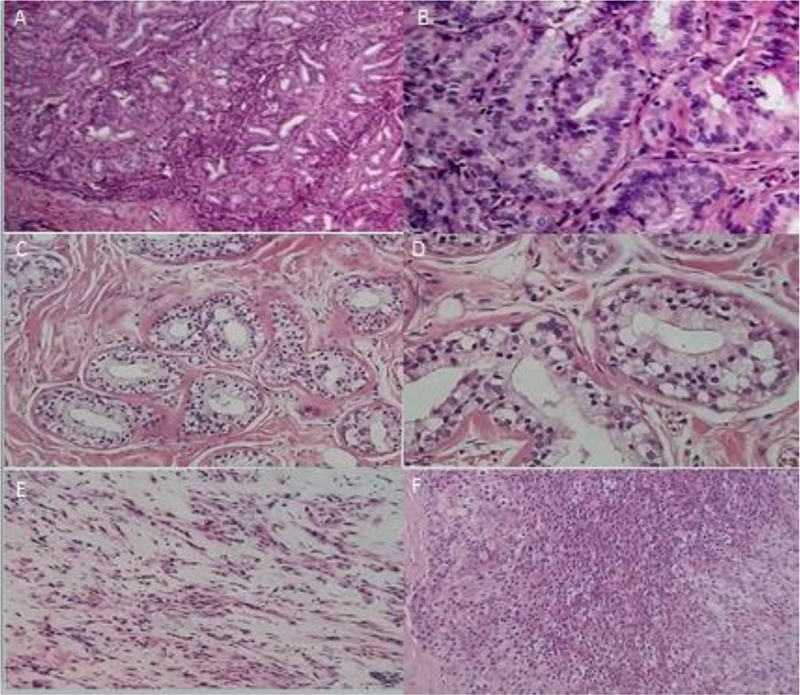
Breast cancer types. **(A)** (×100) and **(B)** (×400), leaflet type. **(C)** (×100) and **(D)** (×400), gland type. **(E)** (×100) and **(F)** (×400), Fibroblast type.

Breast cancer is divided into three types according to the tumor structure and morphology of the myoepithelial. Leaflet type (**Figures 2A,B**): this type was more common, and the tumors had complete or incomplete collagen fiber capsule, the fiber capsule extended to the tumor, and the tumor tissues were divided into nodular and lobular. Hyperplasia of myogenic epithelial cells was solid, nested arranged, and the cytoplasm was bright or acidophilic, and some had around plasmacytoid squeezing the gland cavity. The majority of tumor centers were transparent, and some tumors were calcified with large central infarction. Gland type (**Figures 2C,D**): most of the tumor borders were unclear and characterized by different sizes of the main gland and myoepithelial cells. Glandular epithelial cells were gathered around the ductal hyperplasia, like sclerosing papilloma, tubular and gland tubular adenoma. The duct could be occluded when myoepithelial hyperplasia. Fibroblast type (**Figures 2E,F**): Fibroblast epithelial cells were rich with eosinophilic granules, and myoepithelial cells formed bundle structures. Proliferative cell mass was oppression lumen. The epithelial cells of the gland epithelium were cubic or flat, with a round or oval shape. The cytoplasm was abundant, eosinophilic and common apocrine mucosal secretions. In some areas, fibroids were confused due to the lack of glandular epithelium.

### The Risk Factors of Diarrhea Events

Univariate analysis of potential predictor factors, the risk factors associated with ≥ grade II diarrhea were age, bone and skin metastases, treatment in spring, creatinine and albumin. Grade one diarrhea is also an important risk factor for diarrhea developing into grade II. A negative relation between risk and amount of medical cycles were found. The results showed that diarrhea incidences were highest in the earlier cycle and then decreased by 13% in one more cycle (**Table 4**). Using all the predictors who came out to be significant in the univariate analysis, the following risk factors of diarrhea were analyzed by using a multivariate regression model: each additional cycle of two drugs, skin metastases, grade I diarrhea in prior cycle, therapy started in the spring, and brain metastases. The analysis showed that skin metastases, grade I diarrhea in prior cycle, therapy started in the spring, and brain metastases, were found to be significantly associated with IA development (**Table 5**).

**Table 4:** Predictive factors for diarrhea derived from the database.

Variable	Odds ratio	95% CI	Impact relative risk
Each additional cycle	0.86	0.83–0.98	↓by 13% per cycle
Skin metastases	0.26	0.15–0.78	↓by 73%
Grade I diarrhea in prior cycle	2.6	(0.91–4.7)	↑twofold
Therapy started in the spring	2.3	(1.1–4.2)	↑twofold
Planned dose of capecitabine/cycle (grams)	1.22	(0.91–1.24)	↑by 3.7% with each gram
Brain metastases	6.4	(0.93–38.2)	↑fivefold

**Table 5:** Multivariate model analysis of the risks associated with diarrhea incidences.

Characteristics	*P*-value	Odd ratios	95% CI
Each additional cycle	0.12	1.14	0.64–2.52
Skin metastases	0.04	0.65	0.17–1.69
Grade I diarrhea in prior cycle	0.04	2.38	0.56–7.34
Therapy started in the spring	0.04	2.61	1.07–4.35
Brain metastases	0.02	3.28	1.23–7.68

### EFM Improved Diarrhea and Gastrointestinal Symptoms

**FIGURE 3:**
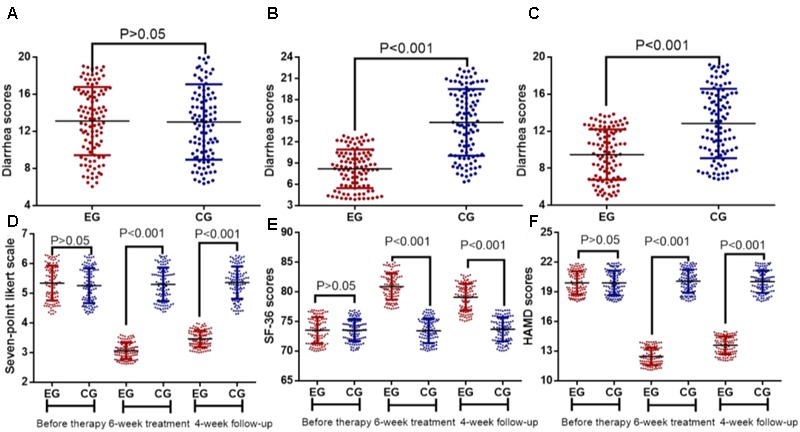
Therapeutic effects of Extracts of *Fructus mume* (EFM) on breast cancer patients with diarrhea. **(A)** diarrhea scores before therapy. **(B)** Diarrhea scores after 6-week therapy. **(C)** Diarrhea scores after 4-week follow-up. **(D)** The effects of EFM on gastrointestinal symptoms. **(E)** The effects of EFM on SF-36 scores. **(F)** The effects of EFM on HADS scores. EG, the patients received EFM treatment. CG, the patients received placebo treatment. *n* = 104 for each group.

The average scores of the diarrhea symptom before EFM treatment were 12.97 ± 6.62 and 13.11 ± 7.53 in EG and CG groups (**Figure 3A**, *P* > 0.05), respectively. After 6-week therapy, the average scores of the diarrhea symptom before EFM treatment were 8.47 ± 4.62 and 14.29 ± 8.15 in EG and CG groups, respectively. The reduction in diarrhea symptoms was statistically significant in EG group when compared with CG group (**Figure 3B**, *P* < 0.001). After further 4-week follow-up, the average scores of the diarrhea symptom before EFM treatment were 9.25 ± 4.63 and 13.01 ± 6.24 in EG and CG groups, respectively. The reduction in diarrhea symptoms was still statistically significant (**Figure 3C**, *P* < 0.001). Similarly, the statistical difference for gastrointestinal symptoms was insignificant between EG and CG groups (**Figure 3D**, *P* > 0.05). After 6-week therapy and 4- week follow-up, the reduction in gastrointestinal symptoms was statistically significant in EG group when compared with CG group (**Figure 3D**, *P* < 0.001).

### EFM Improved Quality of Life

The statistical difference for quality of life was insignificant between EG and CG groups before EFM treatment (**Figures 3E,F**, *P* > 0.05). After 6-week therapy and 4-week follow-up, the improvement of quality of life was statistically significant in EG group when compared with CG group (**Figures 3E,F**, *P* < 0.001).

## Discussion

Diarrhea is a common side effect for the agents targeting MBC patients. The present findings demonstrated that EFM improved, diarrhea and gastrointestinal symptoms, and life quality in HER-2 positive breast cancer patients receiving the combination therapy of lapatinib and capecitabine (**Figure 3**). The main method in the treatment of chronic diarrhea is to use medicine to regulate gastrointestinal motility (Sobczak et al., 2014; Jabri et al., 2016), intestinal flora (Sherman, 2012; Sun et al., 2014) and protect intestinal mucosal vaccine (Li B. et al., 2017; Zhang et al., 2018). However, most medicine causes unwanted adverse side effects. For example, There is increasing evidence to prove that rifaximin is an effective chemoprophylactic drug against travelers’ diarrhea, especially in the subjects who have high risk of severe complications caused by acute infectious diarrhea (Qi et al., 2017) and inhibited most anaerobic bacteria (Finegold et al., 2009), whereas the patients may develop neutropenia after the administration of rifaximin (Hynicka and Silva, 2012). Tricyclic antidepressant amitriptyline has good efficacy in irritable bowel syndrome with diarrhea (IBS-D) (Sohn et al., 2012), whereas vaccine may cause systemic side effects and its safety and tolerability should be carefully considered for its clinical application (Kaka et al., 2017).

Extracts of *F. mume* has been widely accepted drugs for the treatment of various diarrhea with its anti-oxidant and anti-inflammatory properties (Ku et al., 2016), which have repair and defense capabilities for the gastrointestinal tract. Diarrhea can be caused by various etiologies, such as intestinal secretion, absorption of water and destroyed electrolyte, leading to loss of gastrointestinal capacity and osmotic pressure. Therefore, to maintain intestinal cell motility and electrolyte balance, repair and improve the mucosal defense function of the attack factor is an important method in the treatment of diarrhea. Fortunately, EFM have such functions to regulate gastrointestinal motility (Wang et al., 2010) and intestinal secretion (Liu et al., 2009) so on.

Citric acid, 5-HMF, and chlorogenic acid were the main ingredients of EFM. Citric acid has been reported to inhibit the all enveloped and non-enveloped viruses that can cause diarrhea (Ionidis et al., 2016). 5-HMF also has antioxidant, anti- diarrhea, antiviral and antibacterial effects (Kuo et al., 2002). Chlorogenic acid is an antioxidant polyphenol in fruits and vegetables. Chlorogenic acid can prevent intestinal inflammatory conditions (Shin et al., 2015). All these characters of three ingredients may contribute to the protective role of EFM in preventing diarrhea risk.

Diarrhea will contribute to gastrointestinal illness and the effects of EFM on gastrointestinal symptoms were also explored by using seven-point Likert scale. The results suggest the ingredients of EFM may contribute to more desirable gastrointestinal milieu. Patients with diarrhea often have psychiatric comorbidities (Holtmann et al., 2017) and alterations in the intestinal microbiota have been regarded to be associated with depression (Liu et al., 2016). The present study showed that EFM may have anti-depression properties by reducing HADS scores. Diarrhea is also associated with significant impairment in health-related quality of life (Buono et al., 2017). EFM treatment could improve life quality of MBC patients by increasing SF-36 scores.

The present univariate and multivariate analyses showed that skin metastases, grade I diarrhea in prior cycle, therapy started in the spring, and brain metastases were associated with diarrhea severity. All these factors were all consistent with previous reports. Lapatinib treatment may induce diarrhea and skin side effects (Becze, 2010), whereas skin lesions also can increase diarrhea risks (Fallahzadeh et al., 2013). Diarrhea grade 1 does not require treatment but it can induce the risk of diarrhea grade 2 or a higher grade (Brandes et al., 2004). Spring is an important season to control diarrhea (Yong-song et al., 2007). Brain-gut function plays an important role in the diarrhea risk (Zhao et al., 2015) whereas brain metastases will affect the interaction between brain and gut.

There were some limitations in the present stud. The present study mainly focused on only diarrhea and most other parameters were not measured, such as intestinal secretion, absorption of water and destroyed electrolyte. We did not explore the molecular mechanisms for the functional role of EFM in the treatment of diarrhea cause by the combination of lapatinib and capecitabine. Thus, the exact mechanism for the effects of EFM on the diarrhea remains unclear. There are also other safety aspects, including dose intensity, rash, mucositis and drug interactions that were not addressed in the present manuscript, but would be important to consider in the future work. There should be a dose difference among these patients and the related results would be critical to understand functional role of EFM. Therefore, further work is highly demanded to address these issues.

In sum, the results of this study suggest that EFM can significantly improve diarrhea and gastrointestinal symptoms, and the life quality of MBC patients with the diarrhea caused by the combination therapy of lapatinib and capecitabine. EFM may be a potential choice for treating the diarrhea caused by the combination therapy of lapatinib and capecitabine in breast cancer patients.

## Author Contributions

HX designed the experiments. LZ collected all data. JM analyzed all data. ZL and CS performed all the experiments and analyzed the data. YL wrote the paper.

## Conflict of Interest Statement

The authors declare that the research was conducted in the absence of any commercial or financial relationships that could be construed as a potential conflict of interest.
